# Alterations of White Matter Integrity and Hippocampal Functional Connectivity in Type 2 Diabetes Without Mild Cognitive Impairment

**DOI:** 10.3389/fnana.2018.00021

**Published:** 2018-03-20

**Authors:** Qian Sun, Guan-Qun Chen, Xi-Bin Wang, Ying Yu, Yu-Chuan Hu, Lin-Feng Yan, Xin Zhang, Yang Yang, Jin Zhang, Bin Liu, Cong-Cong Wang, Yi Ma, Wen Wang, Ying Han, Guang-Bin Cui

**Affiliations:** ^1^Department of Radiology & Functional and Molecular Imaging, Key Lab of Shaanxi Province, Tangdu Hospital, The Military Medical University of PLA Airforce (Fourth Military Medical University), Xi’an, China; ^2^Department of Neurology, XuanWu Hospital, Capital Medical University, Beijing, China; ^3^Department of Medical Image Diagnosis, Hanzhong Central Hospital, Hanzhong, China; ^4^Student Brigade, The Military Medical University of PLA Airforce (Fourth Military Medical University), Xi’an, China

**Keywords:** type 2 diabetes mellitus (T2DM), functional connectivity (FC), hippocampal, resting-state functional magnetic resonance imaging (rs-fMRI), tract-based spatial statistics (TBSS)

## Abstract

**Aims**: To investigate the white matter (WM) integrity and hippocampal functional connectivity (FC) in type 2 diabetes mellitus (T2DM) patients without mild cognitive impairment (MCI) by using diffusion tensor imaging (DTI) and resting-state functional magnetic resonance imaging (rs-fMRI), respectively.

**Methods**: Twelve T2DM patients without MCI and 24 age, sex and education matched healthy controls (HC) were recruited. DTI and rs-fMRI data were subsequently acquired on a 3.0T MR scanner. Tract-based spatial statistics (TBSS) combining region of interests (ROIs) analysis was used to investigate the alterations of DTI metrics (fractional anisotropy (FA), mean diffusivity (MD), λ_1_ and λ_23_) and FC measurement was performed to calculate hippocampal FC with other brain regions. Cognitive function was evaluated by using Mini-Mental State Examination (MMSE) and Montreal Cognitive Assessment (MoCA). Brain volumes were also evaluated among these participants.

**Results**: There were no difference of MMSE and MoCA scores between two groups. Neither whole brain nor regional brain volume decrease was revealed in T2DM patients without MCI. DTI analysis revealed extensive WM disruptions, especially in the body of corpus callosum (CC). Significant decreases of hippocampal FC with certain brain structures were revealed, especially with the bilateral frontal cortex. Furthermore, the decreased FA in left posterior thalamic radiation (PTR) and increased MD in the splenium of CC were closely related with the decreased hippocampal FC to caudate nucleus and frontal cortex.

**Conclusions**: T2DM patients without MCI showed extensive WM disruptions and abnormal hippocampal FC. Moreover, the WM disruptions and abnormal hippocampal FC were closely associated.

**Highlights**

- T2DM patients without MCI demonstrated no obvious brain volume decrease.
- Extensive white matter disruptions, especially within the body of corpus callosum, were revealed with DTI analysis among the T2DM patients.
- Despite no MCI in T2DM patients, decreased functional connectivity between hippocampal region and some critical brain regions were detected.
- The alterations in hippocampal functional connectivity were closely associated with those of the white matter structures in T2DM patients.

This trial was registered to ClinicalTrials.gov (NCT02420470, https://www.clinicaltrials.gov/).

## Introduction

The latest data released by International Diabetes Federation (IDF) shows that the global number of adults with diabetes in 2015 was 415 million, and is expected to reach 642 million by 2040; In China, that number of diabetic patients is more than 100 million now, of which type 2 diabetes mellitus (T2DM) accounts for 90% (International Diabetes Federation, 2015). T2DM is a major risk for cardiovascular disease, cancer, cerebral complications (Geijselaers et al., 2015) and also associated with cognitive impairment (Benedict et al., 2012; Reijmer et al., 2013) which usually manifests as progressive loss of memory, execution function and information process speed. A significant portion of T2DM patients with cognitive impairment eventually progress to dementia (Crane et al., 2013; Mayeda et al., 2015).

Mild cognitive impairment (MCI) is the early stage of diabetic cognitive impairment (Hunderfund et al., 2006). Previous studies have showed significant brain abnormalities in MCI, including the abnormal resting-state functional connectivity (FC) in frontal cortex, temporal cortex and even the whole brain (Wang et al., 2016), abnormal amplitude of low frequency fluctuations (ALFF) in the precuneus and posterior cingulate cortex (Xi et al., 2012), abnormal regional homogeneity (ReHo) in the temporal and occipital cortex (Yuan et al., 2016). Meanwhile, T2DM patients with MCI were reported with decreased total gray matter (GM) volume, and middle temporal cortex volume was found to be associated with the increased MCI risk in T2DM patients (Zhang Y. et al., 2014). All these brain abnormalities were suggested to be the potential imaging biomarkers for MCI in T2DM patients. However, the occurrence of MCI was an irreversible process, making pre-MCI stage (T2DM patients without MCI) the best time-window of intervening T2DM patients. In view of this, it is of significance to explore whether the brain structure or function has been affected in T2DM patients without MCI. If yes, it could potentially establish early biomarkers in T2DM patients before they develop cognitive impairment and in-time interventions can be delivered to prevent or delay cognitive impairment. Considering this, whole brain or regional atrophy are not ideal biomarkers since they usually occur when T2DM patients already developed MCI.

According to previous studies, long-term hyperglycemia leads to white matter (WM) impairment (Yang et al., 2011; van Bloemendaal et al., 2016; van Bussel et al., 2016) and brain dysfunction (Xia et al., 2013; Wang et al., 2016). Utilizing diffusion tensor imaging (DTI) technique, extensive WM disruptions had been revealed and were correlated with MCI in T2DM patients (Reijmer et al., 2010; Zhang J. et al., 2014). On the other hand, resting-state functional magnetic resonance imaging (rs-fMRI; Musen et al., 2012; Xia et al., 2013; Chen et al., 2014; Cui et al., 2015) was used to reveal certain abnormal functional alterations within several functional networks in T2DM patients with MCI, especially default mode network (DMN), attention network and visual network (Musen et al., 2012; Chen et al., 2014; Cui et al., 2015; Xia et al., 2015). Specially, hippocampal structures play critical roles in learning and memory function (Dou et al., 2005; Comijs et al., 2009; Zhou et al., 2010; Tuligenga et al., 2014), and were quite vulnerable to blood glucose fluctuations (Yau et al., 2014), due to the enriched glucose receptors in these regions (Dou et al., 2005). Aberrant hippocampal FC with other brain regions have been demonstrated in T2DM patients (Zhou et al., 2010). However, it remains unclear whether there are detectable WM and hippocampal FC alterations in T2DM patients without MCI.

The purpose of this study was to investigate: (1) the WM integrity and hippocampal FC in T2DM patients without MCI by using DTI and rs-fMRI; and (2) the relationship between WM integrity and hippocampal FC in these patients.

## Materials and Methods

### Subjects

This study was carried out in accordance with the recommendations of “Medical research Ethics Committee of XuanWu Hospital, Capital Medical University” with written informed consent from all subjects. All subjects gave written informed consent in accordance with the Declaration of Helsinki.

All T2DM patients were diagnosed by a physician and had a history of using oral antidiabetic medications or insulin. All subjects underwent a series of standardized clinical evaluations. The duration of diabetes was calculated since the patients’ T2DM diagnosis. Exclusion criteria were: (1) previous history of brain disease, including stroke, epilepsy, trauma, surgery, transient ischemic attack tumors, infarction, or hemorrhage; (2) severe hypertension, i.e., systolic pressure of ≥140 mmHg, or diastolic pressure of ≥90 mmHg after taking antihypertensive prior to MRI scanning; (3) systemic disease such as severe anemia, thyroid dysfunction, syphilis, or acquired immune deficiency syndrome; (4) alcohol or drug abuse; and (5) contraindications for MRI scan, such as pacemaker, prosthetic heart valve, or claustrophobia.

### Neuropsychological Testing

All participants underwent neuropsychological tests including the Chinese version of Mini-Mental State Examination (MMSE) and Beijing version of Montreal Cognitive Assessment (MoCA). In this study, the MMSE was used to screen dementia (Li et al., 2016) and the MoCA was used to evaluate MCI (Lu et al., 2011). All subjects showed normal performance on both MMSE and MoCA (Table 1).

**Table 1:** Demographic and clinical characteristics in type 2 diabetes mellitus (T2DM) and healthy control (HC) groups.

	T2DM	HC	*t*/*χ*^2^/*F*	*P*-value
Age (years)				
Mean ± SD	67.33 ± 4.72	66.67 ± 5.42	0.36	0.719
Range	60–74	57–76		
Gender (male/female)	4/8	8/16	0.00	1.000*
Education (years)	12.00 ± 5.19	11.17 ± 4.82	0.48	0.637
Diabetes duration (years)	7.92 ± 5.30	N/A	N/A	N/A
BMI (kg/m^2^)	25.11 ± 2.55	23.94 ± 3.84	0.95	0.349
Hypertension (yes/no)	6/6	18/6	2.25	0.157
MMSE	28.00 ± 2.00	27.83 ± 2.60	0.04	0.840^#^
MoCA	26.83 ± 2.33	26.04 ± 3.70	0.25	0.624^#^

Values are means ± SD (or frequency). T2DM, type 2 diabetes mellitus; HC, healthy controls; BMI, body mass index; MMSE, mini-mental state examination; MoCA, Montreal cognitive assessment scale. The comparisons of demographics and clinical characteristics between the two groups were obtained using two-sample t-test. *The P value for gender distribution in the two groups was performed using a χ^2^ test. ^#^The differences in neuropsychological scores between two groups were tested for significance with ANCOVA adjusted for age, sex and education. *P* < 0.05 was considered significant.

### Image Acquisition

MRI scan was performed on a 3.0 Tesla Magnetom Trio Tim scanner (Siemens, Erlangen, Germany) using an 8-channel phased-array head coil. All participants underwent the 3D T1-weighted magnetization-prepared rapid gradient echo (3D-MP-RAGE) scan. The scanning parameters were: repetition time (TR) = 1900 ms, echo time (TE) = 2.2 ms, flip angle (FA) = 9°, matrix = 256 × 256, field of view (FOV) = 256 × 256 mm^2^, sagittal slices = 176, thickness = 1 mm.

DTI data were obtained using single shot spin echo-echo planar imaging (SS-SE-EPI) sequence in 30 independent, non-collinear directions of b = 1000 and 0 s/mm^2^ with the following parameters: TR = 11,000 ms, TE = 98 ms, FA = 90°, matrix = 128 × 128, FOV = 256 × 256 mm^2^, slices = 60, thickness = 2 mm.

Rs-fMRI images were collected by using an echo-planar imaging (EPI) sequence, with the following parameters: TR = 2000 ms, TE = 40 ms, FA = 90°, matrix = 64 × 64, slices = 28, thickness = 4 mm, gap = 1 mm. All subjects were asked to close their eyes, lie down, relax, stay awake and try not to think about anything during data acquisition.

### Image Analysis

#### Voxel-Based Morphometry (VBM) Analysis

3D-MP-RAGE images preprocessing was performed with Statistical Parametric Mapping 8 (SPM8^1^; Ashburner, 2009). First, images were segmented into GM, WM and cerebrospinal fluid (CSF) with a bias field correction. Diffeomorphic Anatomical Registration Through Exponentiated Lie Algebra (DARTEL) was used to create a group-specific GM and WM template as well as individual flow fields. These flow fields contained the non-linear deformations between each individual’s MRI-scan and the DARTEL template. The individual GM and WM segmentations were registered to Montreal Neurological Institute (MNI) standard space with linear affine registration and nonlinear deformation using the flow fields. The images were modulated to preserve the relative volume and corrected for brain size. Finally, the segmented, modulated and normalized images were smoothed using a 6 mm full width at half maximum (FWHM) Gaussian kernel. The two-sample *t*-test was conducted between the two groups. The significance threshold for between-group differences was set at *P* < 0.05, False Discovery Rate (FDR) corrected for multiple comparisons.

#### DTI Analysis

DTI processing was performed using FMRIB’s Diffusion Toolbox of FMRI’s Software Library (FSL^2^; Woolrich et al., 2009). First, DTI scans and gradient-vectors were corrected for motion and eddy-current induced distortions; Next, based on the eigenvalues of the tensor, fractional anisotropy (FA), mean diffusivity (MD), axial diffusivity (λ_1_), and radial diffusivity (λ_23_) values were calculated for each voxel. Tract-Based Spatial Statistics (TBSS) analysis was applied for voxel-wise statistical analysis (Smith et al., 2006). Using FSL’s nonlinear image registration algorithm, all subjects’ FA maps were aligned into FMRIB58_FA standard space. Then a mean FA image was created and was thinned to create a mean FA skeleton. The FSL randomize tool was used to perform permutation-based non-parametric inference on the skeleton FA data at a threshold of 0.2 with 5000 permutations. The significance threshold was set at *P* < 0.05, Family Wise Error (FWE) corrected for multiple comparisons. Individual non-linear warps and skeleton projection of FA images were used to project MD, λ_1_, and λ_23_ to the skeleton. The skeletonized FA, MD, λ_1_ and λ_23_ maps were subsequently fed to statistical analysis. John Hopkins University (JHU) WM atlas was used to identify the location of WM tracts that contained the clusters with significant between-group differences and parcellate them into different region of interests (ROIs). Then, the regional DTI metrics were calculated by averaging the corresponding values of each voxel within ROIs.

#### Rs-fMRI Analysis

Rs-fMRI data were preprocessed by using the Data Processing Assistant for Resting-State fMRI (DPARSF) and the SPM8 software package (Chao-Gan and Yu-Feng, 2010). The first 10 time points were discarded, and the remained images were corrected for slice timing and head motion. Scans with head motion of translation >3.0 mm or rotation >3° were excluded. The resulting images were smoothed with 6 mm FWHM isotropic Gaussian kernel. Linear trends were removed from the image time series, and data were band-pass filtered at 0.01–0.08 Hz.

Bilateral hippocampal regions including hippocampus and parahippocampus were selected as seed from Wake Forest University (WFU) pick atlas to perform FC analysis by using rs-fMRI data analysis toolkit (REST)^3^. Pearson correlation coefficients were transformed to standard *z* values using the Fisher r-to-z transformation to improve normal distribution and then stood for the strength of connectivity. One-sample *t*-tests were used to determine brain regions with significant non-zero connectivity to the seed regions, with FWE corrected. Two-sample *t*-tests were performed to compare the hippocampal FC differences between T2DM and HC groups. The threshold was corrected with AlphaSim (*P* < 0.05, minimum cluster size was set to 736 mm^3^ to left hippocampal region and 652 mm^3^ to right hippocampal region).

#### Statistical Analysis

Demographic, clinical, neuropsychological, and behavioral data were analyzed in SPSS 20.0 (SPSS, Chicago, IL, USA). The comparisons of demographic and clinical data between two groups were performed using two-sample *t*-tests or *χ*^2^ test. The differences in neuropsychological scores between the two groups were tested for significance with analysis of covariance (ANCOVA), with age, sex, and education as covariates. Pearson’s correlation analysis between each altered DTI metric values within each WM ROIs and abnormal hippocampal FC values within each GM regions were performed. And then the relationships were further verified by using ANCOVA, with age, sex and education as covariates. The threshold value for establishing significance of group effects was set at *P* < 0.05.

## Results

### Demographic and Clinical Characteristics

There were no significant differences in age, gender, education and body mass index (BMI) between T2DM patients and HC groups (all *P* > 0.05). No significant differences were found in MMSE and MoCA scores between the groups adjusted for age, sex and education (MMSE, *F* = 0.042, *P* = 0.840; MoCA, *F* = 0.245, *P* = 0.624; Table 1). In addition, there were no subjects with MCI in both T2DM and HC groups.

### Brain Volume

**Figure 1:**
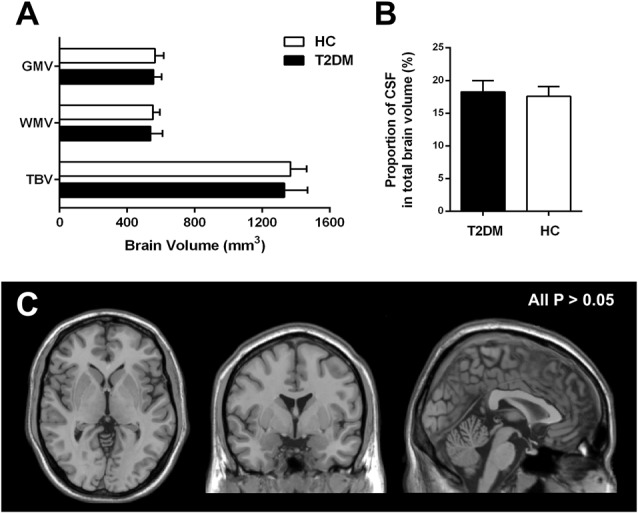
Group difference based on voxel-based morphometry (VBM) analysis. **(A,B)** Difference of the mean gray matter volume (GMV), white matter volume (WMV), total brain volume (TBV) and proportion of cerebrospinal fluid (CSF) in type 2 diabetes mellitus (T2DM) and healthy control (HC) subjects. **(C)** Difference of regional atrophy between T2DM and HC groups. Neither whole brain **(A,B)** nor regional brain **(C)** volume reduction was observed in T2DM patients. All *P* > 0.05.

VBM analyses revealed that there were no significant differences in total GM volume (557.05 ± 48.16 mm^3^ in T2DM vs. 564.81 ± 51.65 mm^3^ in HC, *P* = 0.66), WM volume (539.02 ± 71.20 mm^3^ in T2DM vs. 552.90 ± 40.83 mm^3^ in HC, *P* = 0.54), and total brain volume (TBV; 1330.70 ± 138.07 mm^3^ in T2DM vs. 1367.26 ± 95.53 mm^3^ in HC, *P* = 0.42) between T2DM and HC groups (Figure 1A). Compared with the HC, the proportion of CSF volume in the whole brain was not significantly increased in T2DM patients (18.27 ± 1.72 in T2DM vs. 17.58 ± 1.49 in HC, *P* = 0.22; Figure 1B). When comparing the regional GM, no significant difference was revealed for any specially investigated brain region (Figure 1C).

### WM Integrity

**Figure 2:**
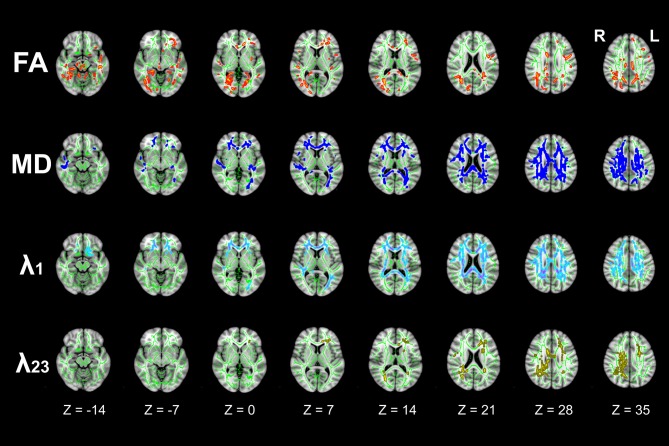
Differences in tract-based spatial statistics (TBSS) analysis results of fractional anisotropy (FA), mean diffusivity (MD), λ_1_, and λ_23_ images between T2DM and HC groups (axial images). Green regions represent the mean FA skeleton of all subjects. Red regions represent tracts with decreased FA, Deep-blue regions represent tracts with increased MD, Light-blue regions represent tracts with increased axial diffusivity (λ_1_), and Yellow regions represent tracts with increased radial diffusivity (λ_23_) in T2DM patients compared with HC subjects (*P* < 0.05, family wise error (FWE) corrected). There were significant differences in extensive white matter (WM) tracts in T2DM patients.

TBSS analyses demonstrated extensive WM impairment in T2DM patients (*P* < 0.05, FWE corrected). Compared to HC, the T2DM patients were with decreased FA, increased MD and λ_1_ in corpus callosum (CC), cingulum bundle, corticospinal tract (CST), internal capsule (IC), external capsule (EC), anterior coronal radiata (ACR), posterior coronal radiata (PCR), fornix, posterior thalamic radiation (PTR), superior cerebellar peduncle (SCP), tapetum etc. In addition, increased λ_23_ was observed in restricted brain regions (Figure 2).

**Figure 3:**
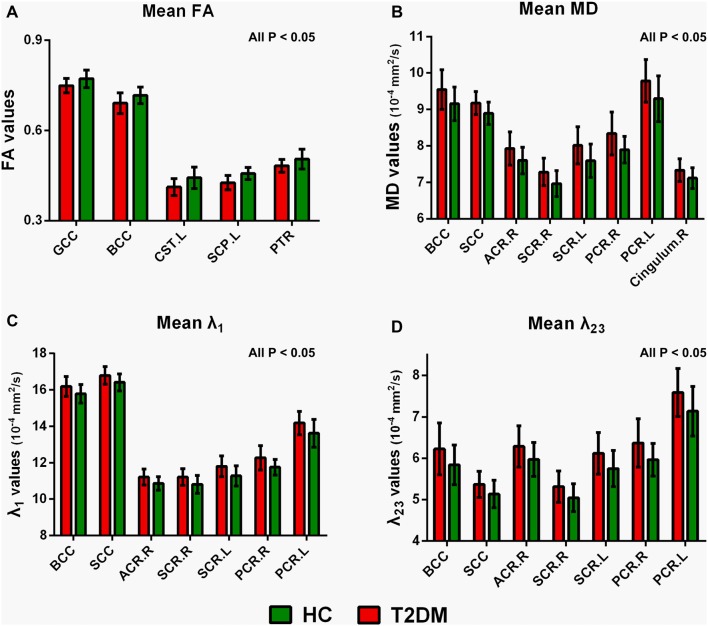
Mean diffusion metrics and group differences of region of interests (ROIs) analysis between T2DM and HC groups. **(A)** Regions with decreased FA in T2DM groups; **(B)** regions with increased MD in T2DM groups; **(C)** regions with increased λ_1_ in T2DM groups; **(D)** regions with increased λ_23_ in T2DM patients. All the ROIs showed in the figure were significantly different (all *P* < 0.05). GCC, genu of corpus callosum; BCC, body of corpus callosum; SCC, splenium of corpus callosum; CST, corticospinal tract. SCP, superior cerebellar peduncle; PTR, posterior thalamic radiation. ACR, anterior corona radiata. PCR, posterior corona radiata. SCR, superior corona radiata. R, right, L, left.

Figure 3 illustrated the mean diffusion metrics of each atlas-based ROIs with significant between-group differences in the two groups. Compared with HC subjects, T2DM patients demonstrated significantly lower FA in body of CC (BCC), genu of CC (GCC), left CST, left SCP and left PTR (Figure 3A); Significantly higher MD in splenium of CC (SCC), BCC, right ACR, bilateral superior corona radiata (SCR), bilateral PCR and right cingulum bundle (Figure 3B); and significantly higher λ_1_ and λ_23_ in BCC, SCC, right ACR, bilateral SCR and bilateral PCR (*P* < 0.05; Figures 3C,D).

### Hippocampal FC

**Figure 4:**
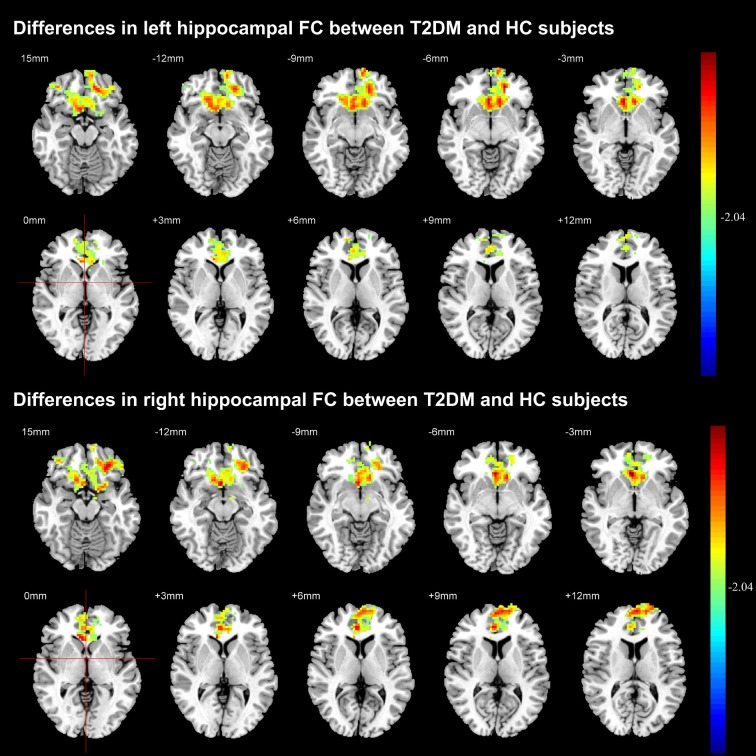
Significant difference in the functional connectivity (FC) of hippocampal region between T2DM and HC groups. The color scale represents the strength of hippocampal FC (decreasing strength from green to red) in axial images. The threshold was set as *P* < 0.05, AlphaSim corrected.

Figure 4 illustrated the decreased hippocampal FC in whole brain. Compared with HC subjects, FC significantly decreased between left hippocampal region and the anterior cingulate cortex, olfactory cortex, caudate nucleus and frontal cortex (including dorsolateral, medial and orbital part of superior frontal gyrus, gyrus rectus, orbital part of middle and inferior frontal gyrus) in T2DM patients. The FC analysis between right hippocampal region and the rest brain regions also achieved similar results (*P* < 0.05, AlphaSim corrected). The right hippocampal FC coefficient decreased mainly in frontal cortex.

### Correlations Between WM Integrity and Hippocampal FC

**Figure 5:**
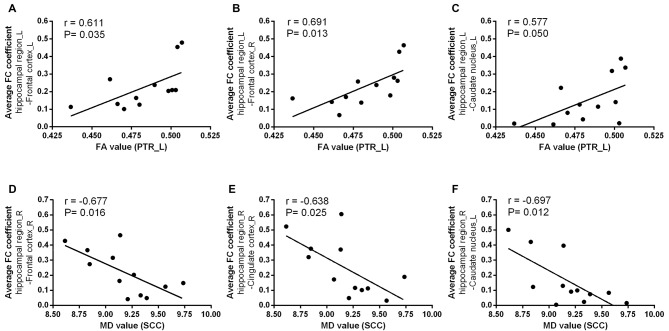
The relationships between WM diffusion metrics and hippocampal FC measures in T2DM. The mean FA values in PTR_L was positively associated with the average coefficient of FC between the left hippocampal region and **(A)** left frontal cortex; **(B)** right frontal cortex; **(C)** left caudate nucleus; The mean MD values in SCC was negatively associated with the average coefficient of FC between the right hippocampal region and **(D)** right frontal cortex; **(E)** right cingulate cortex; **(F)** left caudate nucleus. R, right; L, left. r value means Pearson’s correlation coefficient. *P* value < 0.05 was considered to be statistically significant.

We next examined the relationships between diffusion metrics of ROIs with significant group effects and bilateral hippocampal FC coefficients of GM regions with significant group differences in two groups. In T2DM patients, the mean FA values of left PTR were positive correlated with FC coefficients between the left hippocampal region and left frontal cortex (*r* = 0.611, *P* = 0.035), right frontal cortex (*r* = 0.691, *P* = 0.013) and left caudate nucleus (*r* = 0.577, *P* = 0.050; Figures 5A–C); the mean MD values of SCC were negatively correlated with FC between right hippocampal region and right frontal cortex (*r* = −0.677, *P* = 0.016), right cingulate cortex (*r* = −0.638, *P* = 0.025), and left caudate nucleus (*r* = −0.697, *P* = 0.012; Figures 5D–F). The above correlations were still maintained after the partial correlation analysis, using age and gender as covariates. In addition, there were no significant correlation between WM and FC in the HC group.

## Discussion

In the current study, we reported that T2DM patients without MCI demonstrated: (1) no significant brain volume decrease; (2) extensive WM impairments and abnormal hippocampal FC; and (3) the altered WM integrity were significantly correlated with the decreased hippocampal FC. Our study provided evidence that the WM integrity and hippocampal FC are vulnerable to T2DM at the early stage when patients do not develop cognitive impairment.

A meta-analysis showed that T2DM was associated with atrophy of the whole brain, especially the hippocampal regions (Moulton et al., 2015). In the current study, neither the whole brain (including WM, GM and CSF), nor certain brain region showed significant volume decrease in T2DM patients without MCI. This may be caused by the different disease severity of the target subjects, i.e., T2DM patients in our study were at the early stage of disease compared with other studies that the included T2DM patients were at the late stage with the development of MCI accompanied with brain atrophy (Moran et al., 2013; Roberts et al., 2014). With the disease progress, brain atrophy, especially hippocampal atrophy, may be detected when cognitive impairment occurs in T2DM patients.

Similar to neuroimaging studies of MCI in Alzheimer’s disease (AD) patients (García-Casares et al., 2016), DTI analysis suggested a close relationship between cognitive impairments and WM abnormalities of some specific WM tracts, including the superior longitudinal fasciculus (SLF), the uncinate fasciculus (UF), the inferior longitudinal fasciculus (ILF), and the genu and splenium of the CC, and, especially for the decreased information-processing speed and worse memory performance in T2DM patients (Reijmer et al., 2013). Furthermore, extensive WM impairments especially in whole CC, anterior limb of the IC, and EC, were revealed in T2DM patients with cognitive impairment (especially executive dysfunction; Zhang J. et al., 2014). Taking T2DM patients without MCI as our target population, we revealed extensive WM impairments, with the BCC being the most heavily impaired one. CC is the major commissural fiber connecting both hemispheres and plays key role in transferring, integrating and coordinating information between left and right hemispheres (Caille et al., 2005). BCC, as an important part of CC, connects the bilateral posterior frontal and parietal cortices that contains the important structures of limbic system dealing with memory, emotion and execution as well (Huang et al., 2015). The impairment of BCC implies the actual dysfunction or the vulnerability to dysfunction of memory, emotion and execution (Peltier et al., 2012). These findings in our study suggest that BCC damage is important to understand the cognitive impairment linked to this disease, however, it may not be so specific for T2DM related MCI because varied brain diseases are accompanied with BCC damage.

FA, MD, λ_1_, and λ_23_ are the primary DTI metrics to date to reflect overall WM integrity, maturation, and organization (Reijmer et al., 2013; Zhang J. et al., 2014). Previous studies in T2DM patients have reported different results: Reijmer et al. (2013) showed significantly increased MD in SLF, UF, and ILF in both hemispheres, but T2DM patients showed decreased FA only in UF in the right hemisphere. By contrast, Hoogenboom et al. reported decreased FA in the cingulum bundle and UF, while MD, λ_1_, and λ_23_ showed no obvious change (Hoogenboom et al., 2014). In recent year, Zhang J. et al. (2014) revealed significantly decreased FA, increased MD and λ_23_ in widespread WM tracts in T2DM patients, however, there were no significant tract-specific λ_1_ differences between the two groups. In the current study, we found the whole DTI metrics, including FA, MD, λ_1_, and λ_23_, were significantly different between T2DM and control groups. Accordingly, the alteration of FA and MD might be due to the increased λ_1_ and λ_23_. Given that λ_1_ is related to axonal injury and λ_23_ is related to myelin alteration, our results support that both axonal injury and demyelination are important contributing factor to WM impairments in T2DM patients.

Previous studies demonstrated that T2DM developed aberrant FC of the posterior cingulate cortex (Chen et al., 2014), DMN, left frontal parietal network, sensorimotor network (Chen et al., 2015), as well as attention network (Xia et al., 2015). Hippocampal is particularly vulnerable to long-lasting hyperglycemia (Yau et al., 2014) that usually leads to cognitive impairment (Gold et al., 2007; Hayashi et al., 2011; van Bussel et al., 2016). However, the effect of hyperglycemia on the hippocampal FC in patients without cognitive impairment has not been extensively studied. In the current study, we revealed the decreased hippocampal FC mainly relates with the frontal cortex in T2DM patients without MCI. Based on previous conclusions that the impaired front-temporal connection is closely related with cognitive dysfunction (Gold et al., 2007), our results suggested that the decreased hippocampal FC with frontal cortex may serve as early biomarker to detect vulnerable individuals from T2DM patients.

In addition, we found that the mean FA value of the left PTR were positively related to the decreased FC between the left hippocampal region and bilateral frontal cortex as well as the left cingulate cortex; the mean MD values of SCC were negative correlated with decreased FC between right hippocampal region and right frontal cortex, right cingulate cortex and left caudate nucleus. These findings imply the potential connections between brain structure and brain function and the functional alterations are accompanied with the structural ones. These findings were also consistent with previous neurobiological concepts on the structural basis for brain functions. However, causal relationship between the structural and function alterations needs to be further elucidated in future studies.

There are several limitations for this study. First, only 12 T2DM patients without MCI were recruited. To obtain more accurate and reliable results, more patients should be included in the future. Second, including a number of subjects with hypertension may affect the conclusion. Third, we didn’t re-group the subjects by sex for analysis. In future studies, we will recruit more subjects and re-group them to find out whether there are gender difference in any of the studied parameters. Finally, it must be kept in mind that there are some limitations about the technique itself. There are studies reporting that these analysis methods, including VBM (Smith et al., 2007), TBSS (Smith et al., 2006), and rs-fMRI (Lv et al., 2018), generate spurious results due to imperfect registrations and algorithms.

In conclusion, this study indicates that T2DM patients without MCI show extensive WM disruptions and altered hippocampal FC with frontal cortex. Moreover, these disruptions in WM integrity seem to be closely related with hippocampal FC decrease. The current findings add evidence of the pathophysiological process of MCI in T2DM patients and may suggest structural and functional imaging biomarkers for an early detection of the brain abnormalities that take place before developing the cognitive impairments caused by T2DM.

## Author Contributions

G-BC, YH and WW contributed equally to the conception and design of the study. QS and G-QC contributed equally to this work. G-QC was responsible for data collection. QS, YYu, L-FY, Y-CH, WW, XZ and YYang processed and analyzed the data. X-BW, JZ, BL, C-CW and YM contributed to the data analysis. QS, G-QC, XZ and WW drafted the manuscript. All authors revised and approved the final draft of this article.

## Conflict of Interest Statement

The authors declare that the research was conducted in the absence of any commercial or financial relationships that could be construed as a potential conflict of interest. The reviewer MC-P and handling Editor declared their shared affiliation.

## Footnotes

^1^http://www.fil.ion.ucl.ac.uk/spm

^2^www.fmrib.ox.ac.uk/fsl/

^3^www.restfmri.net
